# Metabolomic differences between critically Ill women and men

**DOI:** 10.1038/s41598-021-83602-5

**Published:** 2021-02-17

**Authors:** Sowmya Chary, Karin Amrein, Jessica A. Lasky-Su, Harald Dobnig, Kenneth B. Christopher

**Affiliations:** 1 Biogen, Inc., 225 Binney St, Cambridge, MA 02142 USA; 2 Division of Endocrinology and Diabetology, Medical University of Graz, Auenbruggerplatz 15, 8036 Graz, Austria; 3 Channing Division of Network Medicine, Brigham and Women’s Hospital, 181 Longwood Avenue, Boston, USA; 4 Thyroid Endocrinology Osteoporosis Institute Dobnig, Jakob-Redtenbachergasse 10, 8010 Graz, Austria; 5 Division of Renal Medicine, Brigham and Women’s Hospital, 75 Francis Street, Boston, 02115 USA

**Keywords:** Metabolomics, Randomized controlled trials

## Abstract

Metabolism differs in women and men at homeostasis. Critically ill patients have profound dysregulation of homeostasis and metabolism. It is not clear if the metabolic response to critical illness differs in women compared to men. Such sex-specific differences in illness response would have consequences for personalized medicine. Our aim was to determine the sex-specific metabolomic response to early critical illness. We performed a post-hoc metabolomics study of the VITdAL-ICU trial where subjects received high dose vitamin D_3_ or placebo. Using mixed-effects modeling, we studied sex-specific changes in metabolites over time adjusted for age, Simplified Acute Physiology Score II, admission diagnosis, day 0 25-hydroxyvitamin D level, and 25-hydroxyvitamin D response to intervention. In women, multiple members of the sphingomyelin and lysophospholipid metabolite classes had significantly positive Bonferroni corrected associations over time compared to men. Further, multiple representatives of the acylcarnitine, androgenic steroid, bile acid, nucleotide and amino acid metabolite classes had significantly negative Bonferroni corrected associations over time compared to men. Gaussian graphical model analyses revealed sex-specific functional modules. Our findings show that robust and coordinated sex-specific metabolite differences exist early in critical illness.

## Introduction

Though inclusiveness of women subjects in clinical research was mandated by the National Institutes of Health (NIH) in 1993, most clinical research studies do not account for sex-specific differences^1–3^. The research that does exist shows that robust differences exist between women and men with respect to disease incidence, disease severity, metabolism and pharmacodynamics of interventions^4,5^. Although firm evidence exists for improved outcomes for female animals in experimental models of severe illness, such differences are not consistently observed in studies on critically ill patients^6–9^. Mechanistic understanding of sex-specific differences in the response to illness is essential if we are to progress to personalized medicine^10^.

Existing data show that metabolism differences are present in healthy women relative to men. At homeostasis, women incorporate free fatty acids into triglycerides whereas men oxidize circulating free fatty acids^11^. Circulating acylcarnitines which are reflective of energy metabolism, are generally lower in women^12^. Women also have less free fatty acid-induced insulin resistance^13^. Healthy women have increases in circulating lipid sphingomyelins which act in cell signaling and may reflect glucose metabolism^14–17^. Sex-specific differences in lipid and cholesterol metabolism are well established and likely due to sex chromosome and sex-specific hormone action^18^. The overall sex-specific metabolism differences at homeostasis are probably due to variation in metabolism related gene expression which contributes to sexual dimorphism^12,19^.

Metabolomics provides a window into the large number of circulating substrates and products of patient’s cellular metabolism^20^. A few large metabolomics studies on healthy individuals are notable for robust metabolite differences related to sex^12,19,21–23^. Data from healthy subjects has little relevance to critically ill patients where metabolic homeostasis is profoundly disturbed^24^. Heterogenous critical illness is not defined by a precise phenotypic framework and studies have provided limited mechanistic insights into pathophysiology^25^. Metabolomic studies performed early in critical illness can reflect illness severity and predict outcomes. But such work does not address sex-specific differences in the response to critical illness^26–28^. Therefore, to see whether sex-specific metabolism differences apply to the critically ill, we studied differences between women and men with regards to changes in metabolism during critical illness.

To test the hypothesis that significant sex-specific plasma metabolomic profile differences exist in the response to critical illness, we performed a metabolomics analysis of 1215 plasma samples from 428 subjects collected during the VITdAL-ICU trial^29^. The VITdAL-ICU trial randomized 492 critically ill adults (166 of whom were women) with 25-hydroxyvitamin D [25(OH)D] levels ≤ 20 ng/ml to high dose oral vitamin D3 or placebo. The VITdAL-ICU trial did not find significant differences in length of hospital stay or mortality outcomes. We assessed the effect of sex on changes in individual metabolites and plasma metabolite families over three time points early in the course of critical illness. Further, with the metabolite change data we determined if regulated metabolite modules exist that associate with sex.

## Results

In the 428 subject analytic cohort, 35% of subjects were women. Baseline characteristics were balanced between subjects stratified by sex for C-reactive protein, Simplified Acute Physiology Score (SAPS) II, day 0 25(OH)D levels, intervention status and ICU type. Differences existed by sex with respect to age (see Table 1 and Supplementary Table S1). The overall 28-day mortality of the 428 subject analytic cohort was 22.2%. The 28-day mortality in women was 22.5% and in men was 22.0%.

**Table 1:** Cohort characteristics.

Characteristic	Female	Male	Total	P-value
No	151	277	428	
Age years Mean (SD)	68.2 (13.3)	62.0 (15.3)	64.2 (14.9)	< 0.001*
Day 0 25(OH)D ng/ml Mean (SD)	13.2 (5.7)	14.4 (10.1)	13.9 (8.8)	0.17*
SAPS II Mean (SD)	34.6 (14.7)	32.7 (15.8)	33.4 (15.4)	0.24*
Day 0 C-reactive protein μg/mL Mean (SD)	119.9 (96.4)	127.6 (86.0)	124.9 (89.8)	0.40*
Day 0 Procalcitonin ng/ml Median [IQR]	0.45 [0.14, 1.98]	0.77 [0.20, 3.02]	0.66 [0.17, 2.79]	< 0.001^†^
Vitamin D_3_ Intervention No. (%)	78 (51.7)	134 (48.4)	212 (49.5)	0.52
Change in 25(OH)D ng/ml Mean (SD)	11.3 (18.0)	10.0 (15.5)	10.4 (16.4)	0.43*
ICU				0.22
Anesthesia ICU No. (%)	24 (15.9)	59 (21.3)	83 (19.4)	
Cardiac Surgery ICU No. (%)	42 (27.8)	84 (30.3)	126 (29.4)	
Surgical ICU No. (%)	7 (4.6)	16 (5.8)	23 (5.4)	
Medicine ICU No. (%)	31 (20.5)	59 (21.3)	90 (21.0)	
Neurological ICU No. (%)	47 (31.1)	59 (21.3)	106 (24.8)	

Data presented as No. (%) unless otherwise indicated. *P*-values determined by chi-square unless designated by (*) then *P*-value determined by ANOVA or by (^†^) determined by Kruskal–Wallis test.

### Single time point data

In day 0 plasma samples (N = 428), significant differences exist in 12 individual metabolites (all multiple test-corrected threshold of *P*-value < 8.65 × 10^–5^, − log_10_(*P*) > 4.06) and in metabolomic profiles (CV-ANOVA *P*-value < 0.001) in female subjects relative to males (see Supplementary Table S2). Regarding subject metabolomic profiles, though the multivariable OPLS-DA model had marginal predictability (Q2 = 0.42), the permutation test confirmed the stability and robustness of the model (Q2 intercept of − 0.387) with a negative permutation Q2 intercept indicating model validity (see Supplementary Table S2)^30,31^. Day 0 differences are present with increased individual sphingomyelin species and decreased androgenic steroids in women relative to men (see Supplementary Table S3).

**Figure 1:**
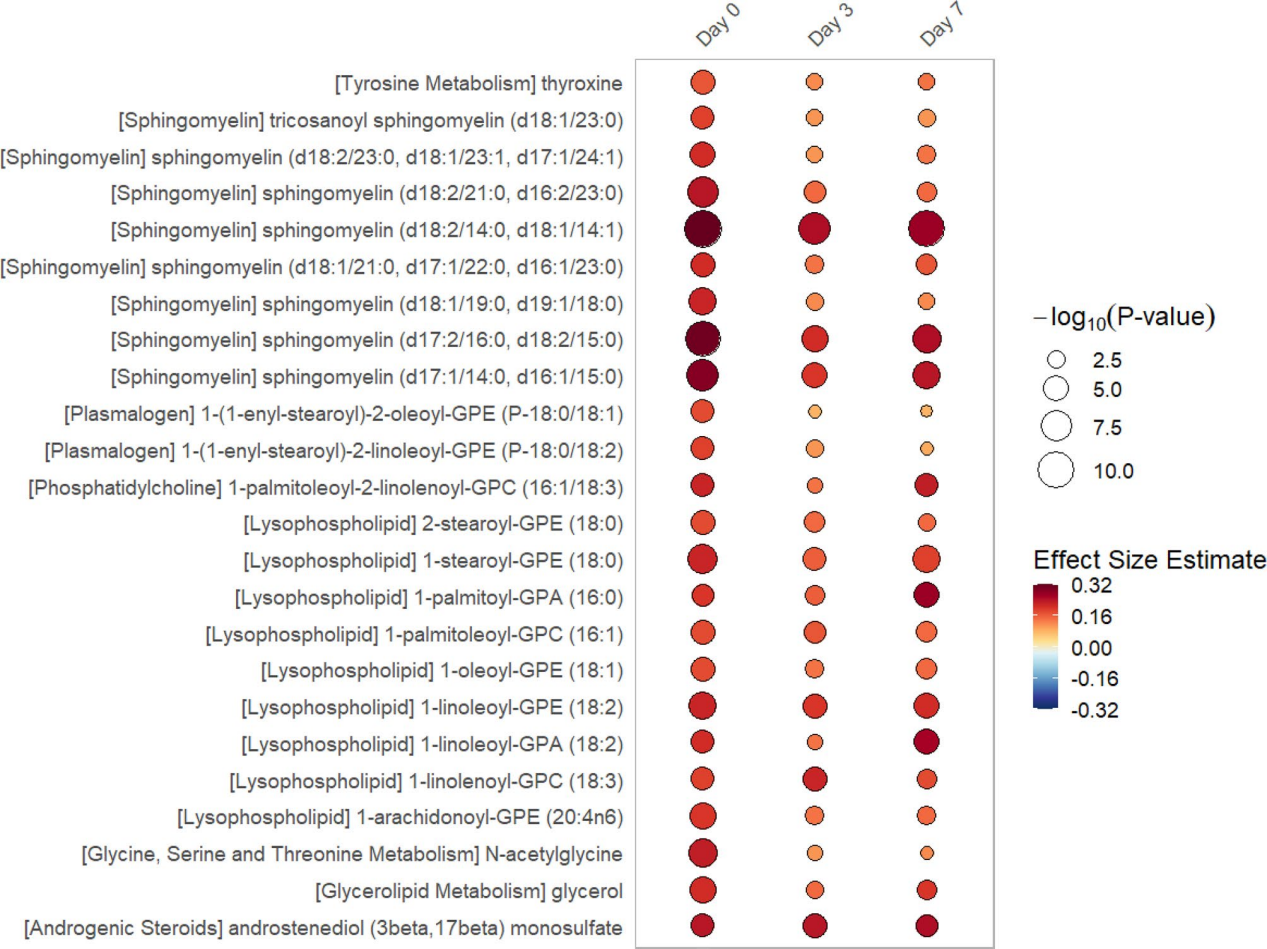
Rain Plot of single time point metabolites Increased in Women. Correlations between individual metabolites and sex at day 0, 3 or 7 were determined utilizing linear regression models correcting for age, SAPS II, admission diagnosis, 25(OH)D at day 0. Day 3 and 7 estimates were also corrected for absolute change in 25(OH)D level at day 3. The magnitude of beta coefficient estimates (effect size) is shown by a color fill scale and the corresponding significance level (− log_10_(*P*-value)) is represented by size of the circle. The intensity of the red fill color represents an increase in effect size for that metabolite in women compared to men. Statistical significance is the multiple test-corrected threshold of − log_10_(*P*-value) > 4.06 which is equivalent to *P*-value < 8.65 × 10^−5^.

**Figure 2:**
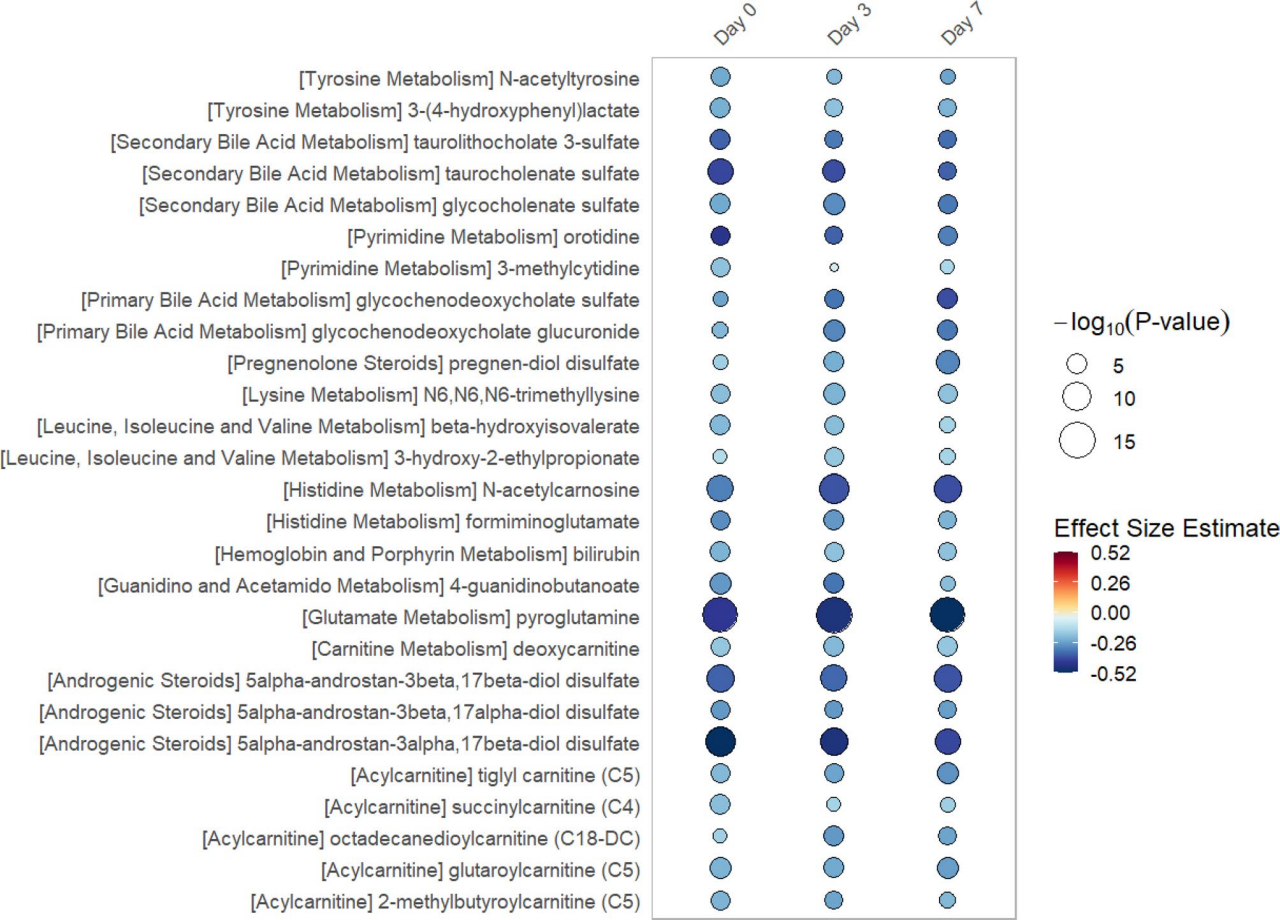
Rain Plot of single time point metabolites Decreased in Women. Correlations between individual metabolites and sex at day 0, 3 or 7 were determined utilizing linear regression models correcting for age, SAPS II, admission diagnosis, 25(OH)D at day 0. Day 3 and 7 estimates were also corrected for absolute change in 25(OH)D level at day 3. The magnitude of beta coefficient estimates (effect size) is shown by a color fill scale and the corresponding significance level (− log_10_(*P*-value)) is represented by size of the circle. The blue fill color represents an decrease in effect size for that metabolite in women compared to men. Statistical significance is the multiple test-corrected threshold of − log_10_(*P*-value) > 4.06 which is equivalent to *P*-value < 8.65 × 10^–5^.

In linear regression models of metabolite data from single time points (day 0, 3 or 7), we find significant differences exist in 51 individual metabolites at 1 or more time point (all multiple test-corrected threshold of *P*-value < 8.65 × 10^–5^, − log_10_(*P*) > 4.06). The rain plots^32^ separately show the metabolites that increase (see Fig. 1) or decrease (see Fig. 2) in women relative to men, with greater significance shown by an increase in circle size. In the data from single time points, significant increases in individual sphingomyelin species and lysophopholipids are found in women when compared to men. Decreases in androgenic steroids as well as bile acid and amino acid metabolism are found in women relative to men.

### Multiple time point data

**Figure 3:**
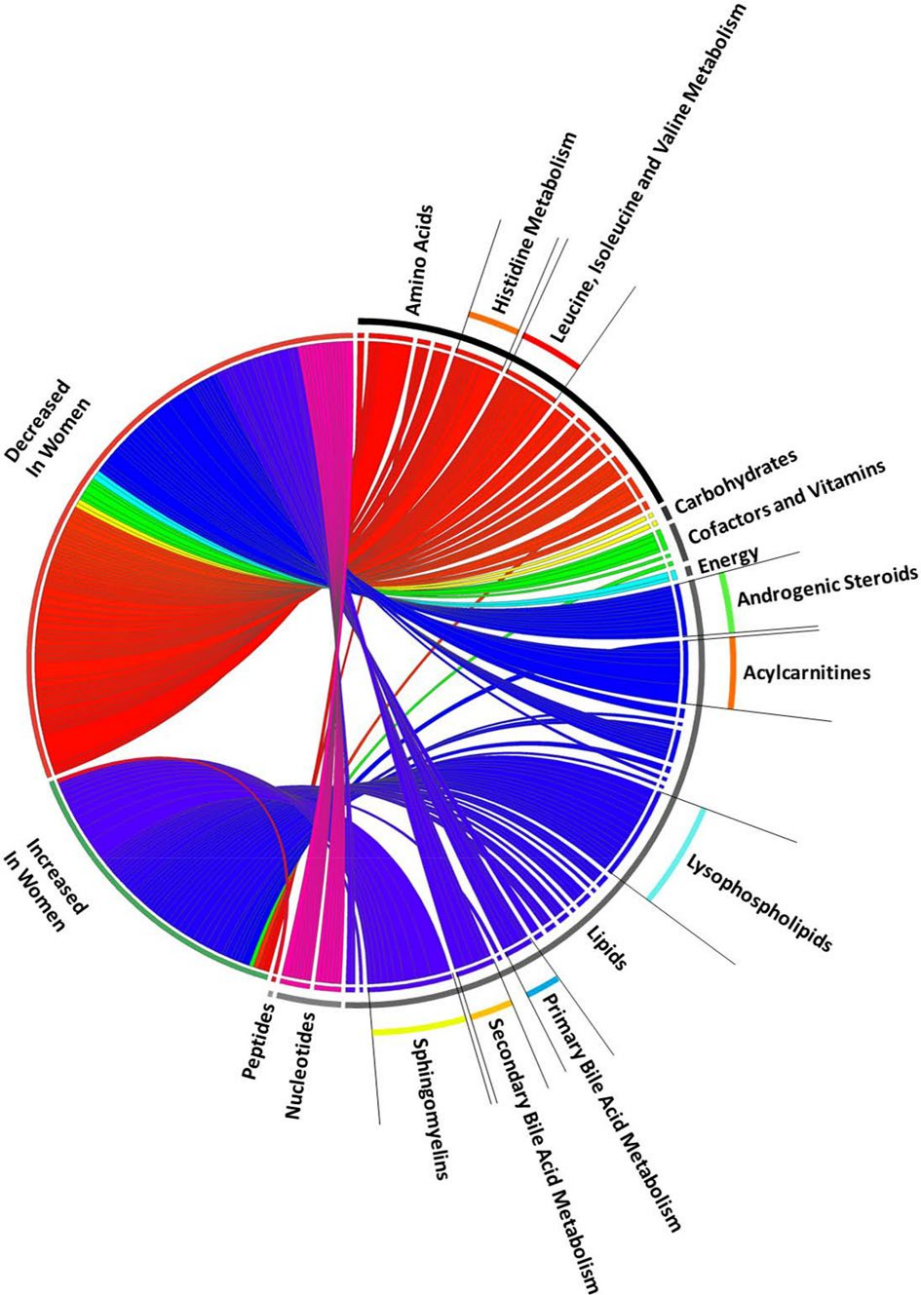
Circos Plot of metabolites over multiple time points. Bipartite graph of metabolites measured in 1215 plasma samples from 428 subjects. Metabolites shown are determined by mixed-effects linear regression to be significantly increased or decreased in women relative to men over the first seven days following trial enrollment. The graph connects the increase or decrease in metabolite on the left side with individual metabolites on the right side. Width of curves indicates strength of the significance (− log_10_(*P*-value)) as determined by mixed-effects regression. Colors differ for each sub-pathway (i.e. all amino acid metabolites are red, all lipids are blue). All curves shown have *P*-value < 8.65 × 10^–5^ in mixed-effects linear regression analysis.

In the repeated measures data, mixed-effects modeling of 1215 total day 0, 3 and 7 plasma samples from the analytic cohort (N = 432) shows 50 metabolites had significantly positive associations in women relative to men highlighted by increases in individual sphingomyelin species and lysophospholipids (see Summarized data in Table 2, Full data in Supplementary Table S4). One hundred five metabolites had significantly negative associations in women relative to men primarily by decreases in acylcarnitine, androgenic steroid, bile acid, nucleotide and amino acid metabolites (see Summarized data in Table 3, Full data in Supplementary Table S5). The mixed-effects modeling of only those subjects who received placebo (N = 216), though limited in power, showed similar patterns as the analytic cohort (N = 432) with Benjamini–Hochberg adjustment^33^ (Supplementary Data 1). data A bipartite graph^34^ highlights metabolites of the lysophospholipid, acylcarnitine, androgenic steroid, bile acid, nucleotide and amino acid metabolite sub-pathways and individual sphingomyelin species that significantly increase or decrease in women relative to men over days 0, 3 and 7 (see Fig. 3).

**Table 2:** Metabolites significantly increased in women relative to men over time.

Metabolite	*P*-value	Bonferroni corrected *P*-value	− log10p	β coefficient	Super pathway	Sub pathway
1-linoleoyl-GPE (18:2)	3.71 E−15	2.14 E−12	14.43	0.21	Lipid	Lysophospholipid
1-palmitoyl-GPA (16:0)	1.77 E−11	1.02 E−08	10.75	0.20	Lipid	Lysophospholipid
1-linoleoyl-GPA (18:2)	5.69 E−10	3.29 E−07	9.24	0.20	Lipid	Lysophospholipid
1-linolenoyl-GPC (18:3)	1.57 E−11	9.08 E−09	10.80	0.20	Lipid	Lysophospholipid
1-stearoyl-GPE (18:0)	6.74 E−16	3.90 E−13	15.17	0.19	Lipid	Lysophospholipid
1-palmitoleoyl-GPC (16:1)	5.95 E−11	3.44 E−08	10.23	0.17	Lipid	Lysophospholipid
1-arachidonoyl-GPE (20:4n6)	2.74 E−10	1.58 E−07	9.56	0.16	Lipid	Lysophospholipid
2-stearoyl-GPE (18:0)	2.13 E−09	1.23 E−06	8.67	0.16	Lipid	Lysophospholipid
1-oleoyl-GPE (18:1)	5.81 E−10	3.36 E−07	9.24	0.16	Lipid	Lysophospholipid
2-palmitoyl-GPC (16:0)	7.59 E−09	4.39 E−06	8.12	0.15	Lipid	Lysophospholipid
1-lignoceroyl-GPC (24:0)	3.89 E−06	2.25 E−03	5.41	0.15	Lipid	Lysophospholipid
1-linoleoyl-GPI (18:2)	9.30 E−08	5.37 E−05	7.03	0.14	Lipid	Lysophospholipid
1-arachidonoyl-GPC (20:4)	8.26 E−07	4.78 E−04	6.08	0.13	Lipid	Lysophospholipid
Sphingomyelin (d18:2/14:0, d18:1/14:1)	6.93 E-29	4.01 E−26	28.16	0.28	Lipid	Sphingomyelin
Sphingomyelin (d17:2/16:0, d18:2/15:0)	1.33 E−21	7.70 E−19	20.88	0.26	Lipid	Sphingomyelin
Sphingomyelin (d17:1/14:0, d16:1/15:0)	1.92 E−18	1.11 E−15	17.72	0.24	Lipid	Sphingomyelin
Sphingomyelin (d18:2/21:0, d16:2/23:0)	4.11 E−13	2.38 E−10	12.39	0.18	Lipid	Sphingomyelin
Sphingomyelin (d18:1/21:0, d17:1/22:0, d16:1/23:0)	3.76 E−10	2.17 E−07	9.43	0.17	Lipid	Sphingomyelin
Sphingomyelin (d18:1/19:0, d19:1/18:0)	1.28 E−09	7.38 E−07	8.89	0.15	Lipid	Sphingomyelin
Sphingomyelin (d18:2/23:0, d18:1/23:1, d17:1/24:1)	2.74 E−09	1.58 E−06	8.56	0.15	Lipid	Sphingomyelin
Tricosanoyl sphingomyelin (d18:1/23:0)	5.28 E−08	3.05 E−05	7.28	0.13	Lipid	Sphingomyelin
Sphingomyelin (d18:1/25:0, d19:0/24:1, d20:1/23:0, d19:1/24:0)	4.60 E−05	2.66 E−02	4.34	0.11	Lipid	Sphingomyelin

Significant results presented following mixed-effects modeling of each of the 578 individual metabolites measured at day 0, 3 and 7. All estimates adjusted for age, SAPS II, admission diagnosis, 25(OH)D at day 0, absolute change in 25(OH)D level at day 3 and plasma day (as the random-intercept). A multiple test-corrected threshold of *P*-value < 8.65 × 10^–5^ was used to identify all significant associations. GPC is glycerophosphorylcholine; GPE is glycerophosphoethanolamine; GPI is glycosylphosphatidylinositol**.** Positive β coefficient values indicate higher abundance in females relative to males.

**Table 3:** Metabolites significantly decreased in Women relative to Men over time.

Metabolite	*P*-value	Bonferroni corrected *P*-value	− log10p	β coefficient	Super pathway	Sub pathway
N-acetylvaline	2.55 E−05	1.47 E−02	4.59	− 0.10	Amino acid	Leucine, isoleucine and valine metabolism
N-acetylleucine	7.65 E−06	4.42 E−03	5.12	− 0.12	Amino acid	Leucine, isoleucine and valine metabolism
Alpha-hydroxyisocaproate	9.27 E−07	5.36 E−04	6.03	− 0.13	Amino acid	Leucine, isoleucine and valine metabolism
3-hydroxyisobutyrate	1.37 E−06	7.90 E−04	5.86	− 0.13	amino acid	Leucine, isoleucine and valine metabolism
3-hydroxy-2-ethylpropionate	2.06 E−09	1.19 E−06	8.69	− 0.15	Amino acid	Leucine, isoleucine and valine metabolism
2-hydroxy-3-methylvalerate	5.00 E−08	2.89 E−05	7.30	− 0.16	Amino acid	Leucine, isoleucine and valine metabolism
2,3-dihydroxy-2-methylbutyrate	4.77 E−07	2.76 E−04	6.32	− 0.17	Amino acid	Leucine, isoleucine and valine metabolism
Beta-hydroxyisovalerate	4.04 E−12	2.33 E−09	11.39	-0.19	Amino acid	Leucine, isoleucine and valine metabolism
Glycochenodeoxycholate	1.59 E−06	9.18 E−04	5.80	− 0.20	Lipid	Primary bile acid metabolism
Taurocholate	4.19 E−05	2.42 E−02	4.38	− 0.21	Lipid	Primary bile acid metabolism
Glycochenodeoxycholate glucuronide	2.94 E−12	1.70 E−09	11.53	− 0.28	Lipid	Primary bile acid metabolism
Taurochenodeoxycholate	2.20 E−08	1.27 E−05	7.66	− 0.28	Lipid	Primary bile acid metabolism
Glycochenodeoxycholate sulfate	4.26 E−11	2.46 E−08	10.37	− 0.32	Lipid	Primary bile acid metabolism
Glycodeoxycholate sulfate	4.01 E−06	2.32 E−03	5.40	− 0.23	Lipid	Secondary bile acid metabolism
Glycolithocholate sulfate	5.82 E−09	3.36 E−06	8.24	− 0.27	Lipid	Secondary bile acid metabolism
Glycocholenate sulfate	6.22 E−14	3.59 E−11	13.21	− 0.28	Lipid	Secondary bile acid metabolism
Taurolithocholate 3-sulfate	3.11 E−12	1.80 E−09	11.51	− 0.34	Lipid	Secondary bile acid metabolism
Taurocholenate sulfate	1.03 E−16	5.94 E−14	15.99	− 0.38	Lipid	Secondary bile acid metabolism

Significant results presented following mixed-effects modeling of each of the 578 individual metabolites measured at day 0, 3 and 7. All estimates adjusted for age, SAPS II, admission diagnosis, 25(OH)D at day 0, absolute change in 25(OH)D level at day 3 and plasma day (as the random-intercept). A multiple test-corrected threshold of *P*-value < 8.65 × 10^–5^ was used to identify all significant associations. Negative β coefficient values indicate lower abundance in females relative to males.

Next, we explored the sex-specific associations of individual metabolites and 28-day mortality. We compared mixed-effects modeling of a total of 441 day 0, 3 and 7 plasma samples from 151 women in the analytic cohort to mixed-effects modeling of a total of 814 day 0, 3 and 7 plasma samples from 277 men in the analytic cohort. The data show that an increase in short chain acylcarnitines C4–C8 and branched-chain amino acids significantly associate with three fold higher 28-day mortality in women but not men (see Supplementary Table S6, Supplementary Fig. S1).

### Metabolic networks and mediation

We investigated sex-specific metabolic networks by measuring pairwise correlations in metabolites which have similar effects via Gaussian graphical models (GGMs). The GGMs analysis revealed seven sex-specific functional modules at day 3 and seven at day 7 (see Supplementary Tables S7 & S8). Similar to the mixed-effects analyses, metabolism of branched chain amino acids, bile acids, androgenic steroids and lysophospholipids are prominently featured in the sex-specific GGM modules. Metabolites within in each functional module were either increased or decreased in women in unison and had biological or functional similarity. Of note, the sex-specific modules do include some individual metabolites that were not significantly associated with sex in our mixed-effects analysis (see Supplementary Tables S7 & S8: Modules B and E, H, I, K, M).

Finally, we focused on the potential mediation of the relationship between individual metabolite abundance and sex by inflammation status. Mediation analyses in day 3 data revealed no influence of Procalcitonin or of C-reactive protein on the associations between sex and each of the individual 578 metabolites (all *P*-values were > 0.01 using 2000 bootstrap samples).

## Discussion

Although previous work suggests that some sex-specific differences exist in the healthy at homeostasis^35^, our data argue that distinct nuanced alterations in metabolism are present in women and men during critical illness. In our single time point data and our mixed-effects methods analysis, we consistently find robust increases and decreases in groups of metabolites along similar sub-pathways that have conserved function (see Fig. 3, Supplementary Tables S4 & S5). Further, we illustrate how groups of metabolites with similar sex-specific effects form GGM modules which highlight the same sub-pathways as our single time point data and our mixed-effects methods analysis^12^*.* These modules serve to focus potential biological interpretation of our sex-specific metabolomics observations^36^. All three analyses highlight the importance of sex-specific metabolism in critical illness related to branched chain amino acids, bile acids, androgenic steroids and lysophospholipids.

Critically ill patients preferentially catabolize fatty acids and amino acids for mitochondrial energy production. Sex-specific differences exist in the utilization of carbohydrates and lipids as energy. With increased energy needs during cell stress, women preferentially oxidize lipids over carbohydrates while men utilize carbohydrates^37^. Firm evidence exists that female mitochondria have higher oxidative capacity, produce less reactive oxygen species, and preferentially utilize lipids for bioenergetics^38–40^. Elevated circulating even-chain C4–C22 acylcarnitines are shown to be due to incomplete mitochondrial β‐oxidation of fatty acids^41,42^. Studies find that increases in circulating acylcarnitines are common in severe critical illness and are associated with adverse outcomes^27^. To see whether these findings apply to critically ill women, we analyzed a total of 36 acylcarnitine species. We demonstrate that 15 circulating acylcarnitine species are significantly lower in women early in critical illness (see Supplementary Table S5). Our data are consistent with a more efficient fatty acid β-oxidation in critically ill women reflective of a sex-specific difference in mitochondrial response to critical illness*.*

The circulating amino acid pool is supplied by dietary amino acids, endogenous amino acid synthesis and cellular protein turnover^43^. Increases in circulating amino acids during critical illness are due to protein catabolism^44^. Skeletal muscle protein is rapidly metabolized in response to severity of illness to provide substrate for liver gluconeogenesis, immune function support and immunoglobulin synthesis^45^. Further, amino acid catabolism is a source for circulating C3, C4 and C5 acylcarnitines^42^. Our findings of decreases in C3, C4 and C5 acylcarnitines as well as in multiple amino acid metabolite sub-pathways suggest sex-specific protein catabolism and energy substrate utilization during critical illness. Of particular interest, are the GGM modules B and H (Supplementary Tables S7 & S8) which highlight the importance of decrease in branched chain amino acid metabolites in women. In women, we observe a combination of decreases in branch chain amino acid metabolites and in dicarboxylate fatty acids generated from fatty acid omega oxidation as well as in short-chain acylcarnitines C3 and C5 derived from branch chain amino acids (Supplementary Table S5). Such decreases strongly suggest improved mitochondrial function and more complete fatty acid β-oxidation in women relative to men^46,47^.

Our novel observations suggest that critically ill women have greater abundance of individual sphingomyelin species, plasmalogens and lysophospholipids compared to men. Studies show that during cell stress, specific sphingomyelin species regulate the initiation of apoptosis and autophagy^48^. Plasmalogens are known to be endogenous antioxidants that protect the endothelium from oxidative stress injury by controlling toxic oxidation products^49^. Lysophospholipids are signaling molecules that have chemoattractant effects and act to modulate the innate immune response^50,51^. Our data are consistent with the hypothesis that the response to cell stress differs in women and men.

The liver is the essential organ for glucose, protein, amino acid, lipid and cholesterol metabolism. Sex-specific differences in liver metabolism at homeostasis are postulated to be an evolutionary consequence of the metabolic flexibility required for reproduction^10^. Our data argue that bile acid metabolites are decreased in critically ill women. Though sex-specific differences in bile acid synthesis are reported^52^, such differences in bile acid homeostasis are not well characterized^53^. It is shown that cytochrome P450 enzymes are important for bile synthesis ^52^ and regulated in a sex-specific manner^54,55^. Bile acids activate the nuclear receptors farnesoid X receptor, pregnane X receptor and vitamin D receptor as well as the G-protein-coupled receptor TGR5. Such bile acid receptor activation results in gene expression which alters metabolism of bile acids, glucose, lipids, energy and inflammation^56^. As elevation in blood bile acids are common in critical illness^57^, and the synthesis and pool composition of bile acids are sex-specific, such differences have widespread downstream metabolism pathway effects.

Our novel study approach has several strengths. The use of a large number of plasma samples at multiple time points early in critical illness allows for a dynamic overview into sex-specific metabolomics (see Fig. 3). Mixed models are extremely useful for metabolomic data measured at multiple time points as they remove confounding variables with a fixed-effect (age, SAPS II, etc.) and also those with a random-effect (plasma sampling day)^58,59^. Importantly, by adjusting for the absolute change in 25(OH)D level at day 3, we mitigate the effect of the trial intervention on the observed sex-specific metabolomic changes which allow for study of the entire trial cohort increasing sample size and study power^60,61^. Further, our use of clinical trial data allows for modelling and normalization of metabolite abundance via adjustment for subject characteristics^62^. To account for multiple comparisons we utilized a conservative Bonferroni corrected *P*-value < 8.65 × 10^–5^ ^63^. Finally, some of the metabolism differences we observe are known to be sex-specific thus increasing the biological plausibility and relevance of our work.

We do acknowledge potential limitations to our approach. Our VITdAL-ICU trial subject population is heterogenous with sex-specific imbalance in some admission diagnosis categories. Despite multivariable adjustment, our approach is subject to bias and confounding. Though our samples are derived from a randomized controlled trial, our study design is observational thus causal inference may be limited. Our subjects were all white with serum levels of 25(OH)D < 20 ng/ml, thus may not be representative of all critically ill. Our use of CRP as an indicator of inflammation is limited in the in the nine reproductive-aged women under study as CRP is associated with Progesterone and Estradiol levels^64^. The single-center setting may limit generalizability of our findings. It is important to recognize that although the function and biological relevance of a metabolite may be characterized, the clinical significance may not be known. Finally, our study is a hypothesis generating exploratory analysis requiring subsequent confirmation and careful interpretation.

The importance of our study is that it offers a nuanced window into the differential metabolic response to critical illness between women and men. Beyond the known sex-dependent metabolism differences at homeostasis, we find that women respond to critical illness stressors in a dramatically different fashion than men. Our findings on sex-specific differences in metabolism pathways is an essential first step toward understanding how to provide patient-centered personalized medicine in the critically ill.

## Methods

The details of the VITdAL-ICU trial^29^ as well as metabolomic processing and analysis are provided in Supplementary Methods. Briefly, the VITdAL-ICU trial randomized 475 critically ill adult patients to vitamin D_3_ or placebo once at a dose of 540,000 IU followed by 90,000 IU monthly^29^. At VITdAL-ICU trial enrollment, written informed consent was obtained and included permission for plasma specimens to be saved for future research studies^29^. The metabolomics study is considered post-hoc as it was designed following initiation completion of the of the VITdAL-ICU trial. The post-hoc study research protocol was approved by the Partners Human Research Committee Institutional Review Board at the Brigham and Women’s Hospital. All research was performed in accordance with the Declaration of Helsinki.

To generate metabolomic data, a total of 1215 plasma samples from 428 VITdAL-ICU trial subjects at day 0, 413 subjects at day 3 and 374 subjects at day 7 were analyzed using four ultra high-performance liquid chromatography/tandem accurate mass spectrometry methods by Metabolon, Inc^65^. Metabolomic profiling identified 769 metabolites (Supplementary Data 2). We reduced baseline noise by removing metabolites with the lowest interquartile range of variability, leaving 578 metabolites^66^. Metabolomic data underwent cube root transformation and Pareto scaling to normalize the distribution^67^.

For univariate analysis of day 0 data, Student’s t-test was performed to determine if significant sex-specific differences exist using MetaboAnalyst^68^. A Bonferroni multiple testing correction threshold of *P*-value < 8.65 × 10^–5^ was used to identify all significant differences^63^. Day 0 data were also analyzed using orthogonal partial least square-discriminant analysis (OPLS-DA), a supervised method to assess the significance of classification discrimination (SIMCA 15.0 Umetrics, Umea, Sweden). Permutation testing was performed for OPLS-DA model validation^30,31^. Sevenfold cross-validation analysis of variance (CV-ANOVA) was utilized to determine OPLS-DA model significance^31^.

For single time point data, correlations between individual metabolites and sex at day 0, 3 or 7 were separately determined utilizing linear regression models correcting for age, SAPS II, admission diagnosis, 25(OH)D at day 0 and absolute change in 25(OH)D level at day 3. A multiple test-corrected threshold of *P*-value < 8.65 × 10^–5^ was used to identify all significant associations in the single time point data^63^. All linear regression models were analyzed using STATA 14.1MP^69^. Rain plots were produced based on hierarchical clustering in R-3.6.2 adapted from source code published by Henglin et al.^32^.

For repeated measures data, correlations between individual metabolites and sex over time (day 0, 3 and 7) were determined utilizing linear mixed-effects models correcting for age, SAPS II, admission diagnosis, 25(OH)D at day 0, absolute change in 25(OH)D level at day 3 and plasma day (as the random-intercept). This analysis was performed in the analytic cohort (N = 428) with multiple test-corrected threshold of *P*-value < 8.65 × 10^–5^ was used to identify all significant associations. We repeated the analysis in only those subjects who received placebo (N = 216) with Benjamini–Hochberg adjustment of *P*-values^33^. All mixed-effects models were analyzed using STATA 14.1MP^69^. For data visualization purposes, a bipartite graph^34^ utilizing the Circos application (http://circos.ca/) in Perl was generated of metabolites which were significantly changed (increased or decreased) in females relative to males.

Mixed effects logistic regression was used separately in 151 women and in 277 men to estimate the odds of 28-day mortality of individual metabolites adjusted for age, SAPS II, admission diagnosis, 25(OH)D at day 0, absolute change in 25(OH)D level at day 3 and plasma day (as the random-intercept). A multiple test-corrected threshold of P-value < 8.65 × 10^–5^ was used to identify all significant associations in the repeated measures data^63^. All mixed-effects models were analyzed using STATA 14.1MP^69^. We used rain plots^32^ to separately visualize the mortality-dependent effect size and significance of individual metabolites in women and men.

As inflammation is important in response to critical illness, we evaluated a potential mediating effect of Procalcitonin or C-reactive protein on the association between sex and individual metabolite abundance adjusted for age, SAPS II, admission diagnosis, 25(OH)D at day 0, absolute change in 25(OH)D level at day 3. Analyses were performed on each of the 578 metabolites at day 3 using the R package mediation^70^ to obtain bootstrap *P*-values (N = 2000 samples)^71,72^. Significant mediation was present if the *P*-value was < 0.01 and 10% or more of the association was mediated through Procalcitonin or C-reactive protein levels^71,72^.

To identify sex-specific modules from metabolomics data, we estimated Gaussian graphical models (GGMs) for day 3 and 7. Modules serve to reconstruct pathway reactions from metabolomics data. GGMs are determined utilizing partial pairwise Pearson correlation coefficients following the removal of the effects of all other metabolites and covariates^73^. We inferred a sex-specific network for relative metabolite abundance. We included age, SAPS II, admission diagnosis, 25(OH)D at day 0, absolute change in 25(OH)D level at day 3 and plasma day as covariates into the model^74^. Edges between metabolites were allotted if both their Pearson correlations and partial correlations remained statistically significant at *P*-value < 0.05 following Bonferroni correction for 578 metabolites^74^. GGMs were produced using the GeneNet R package, version 1.2.13 in R-3.6.2 adapted from published source code^74^.

## Supplementary Information

Supplementary Information 1.

Supplementary Information 2.

Supplementary Figure S1.

Supplementary Information 3.

Supplementary Tables.

Supplementary Legend.
