# Supplementary material for: Metabolomic differences between critically Ill women and men
Source: Sci Rep. 2021 Feb 17;11:3951. doi: 10.1038/s41598-021-83602-5 (PMC7889607; doi:10.1038/s41598-021-83602-5)
Supplement: Supplementary file 3 — Supplementary Figure S1. [file 41598_2021_83602_MOESM3_ESM.pptx]

## Slide 1
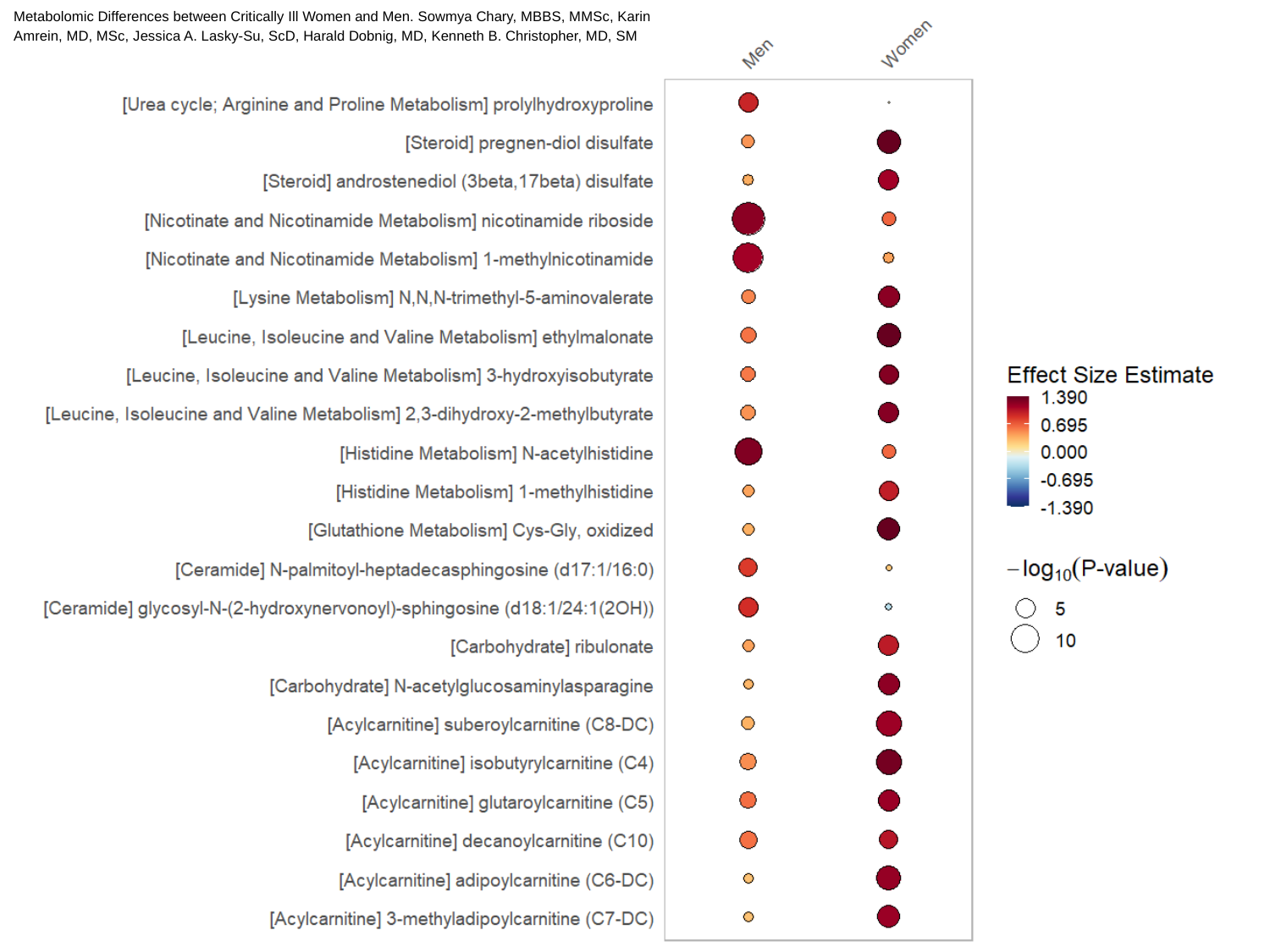

Metabolomic Differences between Critically Ill Women and Men. Sowmya Chary, MBBS, MMSc, Karin Amrein, MD, MSc, Jessica A. Lasky-Su, ScD, Harald Dobnig, MD, Kenneth B. Christopher, MD, SM
