# Supplementary material for: Metabolomic differences between critically Ill women and men
Source: Sci Rep. 2021 Feb 17;11:3951. doi: 10.1038/s41598-021-83602-5 (PMC7889607; doi:10.1038/s41598-021-83602-5)
Supplement: Supplementary file 5 — Supplementary Tables. [file 41598_2021_83602_MOESM5_ESM.docx]

Metabolomic Differences between Critically Ill Women and Men

Sowmya Chary, MBBS, MMSc, Karin Amrein, MD, MSc, Jessica A. Lasky-Su, ScD, Harald Dobnig, MD, Kenneth B. Christopher, MD, SM

**Supplementary Tables**

Supplementary Table S1: Additional Cohort Characteristics

Supplementary Table S2: At randomization (Day 0) OPLS-DA model goodness of fit, predictive ability and goodness of fit and predictive ability

Supplementary Table S3: At randomization (Day 0) Metabolite Differences in Women relative to Men

Supplementary Table S4: Metabolites significantly increased in Women relative to Men over time

Supplementary Table S5: Metabolites significantly decreased in Women relative to Men over time

Supplementary Table S6: Metabolites Differentially associated in women and men with 28-day mortality over time.

Supplementary Table S7: Day 3 Sex-specific Metabolic Networks with similar effects via Gaussian graphical models

Supplementary Table S8: Day 7 Sex-specific Metabolic Networks with similar effects via Gaussian graphical models

**Supplementary Table S1. Additional Cohort Characteristics**

| **Characteristic** | **Female** | **Male** | **Total** |
| --- | --- | --- | --- |
| N | **151** | **277** | **428** |
| Admission Type |  |  |  |
| Brain surgery No. (%) | 2 (1.3) | 2 (0.7) | 4 (0.9) |
| Cardiac surgery No. (%) | 31 (20.5) | 50 (18.1) | 81 (18.9) |
| Cardiovascular No. (%) | 15 (9.9) | 36 (13.0) | 51 (11.9) |
| Gastrointestinal/liver No. (%) | 5 (3.3) | 9 (3.3) | 14 (3.3) |
| Hematologic/oncologic No. (%) | 0 (0) | 1 (0.4) | 1 (0.2) |
| Metabolic/Renal No. (%) | 0 (0) | 8 (2.9) | 8 (1.9) |
| Neurologic No. (%) | 132 (30.3) | 169 (21.3) | 301 (24.5) |
| Other non-operative No. (%) | 2 (0.5) | 6 (0.8) | 8 (0.7) |
| Other operative No. (%) | 17 (3.9) | 17 (2.1) | 34 (2.8) |
| Respiratory No. (%) | 16 (10.6) | 24 (8.7) | 40 (9.4) |
| Sepsis/infectious No. (%) | 11 (7.3) | 24 (8.7) | 35 (8.2) |
| Thoracic surgery No. (%) | 5 (3.3) | 8 (2.9) | 13 (3.0) |
| Transplantation No. (%) | 3 (2.0) | 10 (3.6) | 13 (3.0) |
| Trauma No. (%) | 6 (4.0) | 32 (11.6) | 38 (8.9) |
| Vascular No. (%) | 4 (2.7) | 7 (2.5) | 11 (2.6) |

**Supplementary Table S2. At randomization (Day 0) OPLS-DA model goodness of fit, predictive ability and model significance**

|  | **OPLS-DA** | | | **Permutation (n = 200)** | | **CV-ANOVA** |
| --- | --- | --- | --- | --- | --- | --- |
| Classification Model | R2X | R2Y | Q2 | R2 intercept  (x-axis, y-axis) | Q2 intercept  (x-axis, y-axis) | P-Value |
| Sex | 0.341 | 1.00 | 0.423 | 0.00, 0.385 | 0.00, -0.387 | <0.001 |

**Supplementary Table S3. At randomization (Day 0) Metabolite Differences in Women relative to Men**

| **Metabolite** | **Fold Change** | **Bonferroni corrected P-value** | **-log(10)p** | **Super Pathway** | **Sub Pathway** |
| --- | --- | --- | --- | --- | --- |
| Sphingomyelin (d18:1/25:0, d19:0/24:1, d20:1/23:0, d19:1/24:0) | 3.07 | 7.51 E-10 | 11.89 | Lipid | Sphingomyelin |
| Sphingomyelin (d17:1/14:0, d16:1/15:0) | 2.89 | 1.16 E-09 | 11.70 | Lipid | Sphingomyelin |
| Sphingomyelin (d18:2/14:0, d18:1/14:1) | 2.66 | 4.51 E-07 | 9.11 | Lipid | Sphingomyelin |
| Sphingomyelin (d18:2/21:0, d16:2/23:0) | 2.06 | 4.22 E-04 | 6.14 | Lipid | Sphingomyelin |
| Sphinganine-1-phosphate | 2.52 | 6.94 E-07 | 8.92 | Lipid | Sphingolipid Synthesis |
| 1-palmitoyl-2-oleoyl-GPI (16:0/18:1) | 2.26 | 2.14 E-05 | 7.43 | Lipid | Phosphatidylinositol |
| N-acetylglycine | 2.65 | 3.76 E-05 | 7.19 | Amino Acid | Glycine, Serine and Threonine Metabolism |
| N-acetylcarnosine | 0.44 | 1.27 E-06 | 8.66 | Amino Acid | Histidine Metabolism |
| Pyridoxate | 0.40 | 6.94 E-09 | 10.92 | Cofactors and Vitamins | Vitamin B6 Metabolism |
| 5alpha-androstan-3beta,17beta-diol disulfate | 0.39 | 4.91 E-09 | 11.07 | Lipid | Androgenic Steroids |
| 5alpha-androstan-3alpha,17beta-diol disulfate | 0.39 | 4.91 E-09 | 11.07 | Lipid | Androgenic Steroids |
| Taurocholate | 0.37 | 4.62 E-05 | 7.10 | Lipid | Primary Bile Acid Metabolism |

Note: Fold change greater than 1 indicates an increase in metabolite in women relative to men. A multiple test-corrected threshold of P < 8.65 × 10^-5^ was used to identify all significant differences.

| **Metabolite** | **P-value** | **Bonferroni corrected P-value** | **-log10p** | **β Coefficient** | **Super Pathway** | **Sub Pathway** |
| --- | --- | --- | --- | --- | --- | --- |
| Creatine | 8.96 E-10 | 5.18 E-07 | 9.05 | 0.17 | Amino Acid | Creatine Metabolism |
| N-acetylglycine | 1.02 E-07 | 5.87 E-05 | 6.99 | 0.15 | Amino Acid | Glycine, Serine and Threonine Metabolism |
| Thyroxine | 7.27 E-08 | 4.20 E-05 | 7.14 | 0.14 | Amino Acid | Tyrosine Metabolism |
| Beta-cryptoxanthin | 7.71 E-07 | 4.46 E-04 | 6.11 | 0.17 | Cofactors and Vitamins | Vitamin A Metabolism |
| Androstenediol (3beta,17beta) monosulfate | 3.82 E-12 | 2.21 E-09 | 11.42 | 0.24 | Lipid | Androgenic Steroids |
| N-behenoyl-sphingadienine (d18:2/22:0) | 1.95 E-05 | 1.13 E-02 | 4.71 | 0.11 | Lipid | Ceramides |
| Propionylglycine (C3) | 5.61 E-06 | 3.24 E-03 | 5.25 | 0.14 | Lipid | Fatty Acid Metabolism |
| Hexanoylglycine (C6) | 2.81 E-07 | 1.63 E-04 | 6.55 | 0.17 | Lipid | Fatty Acid Metabolism |
| Glycerol | 7.83 E-10 | 4.53 E-07 | 9.11 | 0.19 | Lipid | Glycerolipid Metabolism |
| Pentadecanoate (15:0) | 3.99 E-05 | 2.31 E-02 | 4.40 | 0.10 | Lipid | Long Chain Fatty Acid |
| 1-linoleoyl-GPE (18:2) | 3.71 E-15 | 2.14 E-12 | 14.43 | 0.21 | Lipid | Lysophospholipid |
| 1-palmitoyl-GPA (16:0) | 1.77 E-11 | 1.02 E-08 | 10.75 | 0.20 | Lipid | Lysophospholipid |
| 1-linoleoyl-GPA (18:2) | 5.69 E-10 | 3.29 E-07 | 9.24 | 0.20 | Lipid | Lysophospholipid |
| 1-linolenoyl-GPC (18:3) | 1.57 E-11 | 9.08 E-09 | 10.80 | 0.20 | Lipid | Lysophospholipid |
| 1-stearoyl-GPE (18:0) | 6.74 E-16 | 3.90 E-13 | 15.17 | 0.19 | Lipid | Lysophospholipid |
| 1-palmitoleoyl-GPC (16:1) | 5.95 E-11 | 3.44 E-08 | 10.23 | 0.17 | Lipid | Lysophospholipid |
| 1-arachidonoyl-GPE (20:4n6) | 2.74 E-10 | 1.58 E-07 | 9.56 | 0.16 | Lipid | Lysophospholipid |
| 2-stearoyl-GPE (18:0) | 2.13 E-09 | 1.23 E-06 | 8.67 | 0.16 | Lipid | Lysophospholipid |
| 1-oleoyl-GPE (18:1) | 5.81 E-10 | 3.36 E-07 | 9.24 | 0.16 | Lipid | Lysophospholipid |
| 2-palmitoyl-GPC (16:0) | 7.59 E-09 | 4.39 E-06 | 8.12 | 0.15 | Lipid | Lysophospholipid |
| 1-lignoceroyl-GPC (24:0) | 3.89 E-06 | 2.25 E-03 | 5.41 | 0.15 | Lipid | Lysophospholipid |
| 1-linoleoyl-GPI (18:2) | 9.30 E-08 | 5.37 E-05 | 7.03 | 0.14 | Lipid | Lysophospholipid |
| 1-arachidonoyl-GPC (20:4) | 8.26 E-07 | 4.78 E-04 | 6.08 | 0.13 | Lipid | Lysophospholipid |
| 1-(1-enyl-oleoyl)-GPE (P-18:1) | 1.09 E-06 | 6.28 E-04 | 5.96 | 0.15 | Lipid | Lysoplasmalogen |
| 1-(1-enyl-palmitoyl)-GPE (P-16:0) | 7.38 E-07 | 4.26 E-04 | 6.13 | 0.14 | Lipid | Lysoplasmalogen |
| 1-(1-enyl-palmitoyl)-GPC (P-16:0) | 1.02 E-06 | 5.91 E-04 | 5.99 | 0.14 | Lipid | Lysoplasmalogen |
| 1-(1-enyl-stearoyl)-GPE (P-18:0) | 5.23 E-06 | 3.02 E-03 | 5.28 | 0.13 | Lipid | Lysoplasmalogen |
| 1-dihomo-linolenylglycerol (20:3) | 1.34 E-05 | 7.72 E-03 | 4.87 | 0.16 | Lipid | Monoacylglycerol |
| 1-palmitoleoyl-2-linolenoyl-GPC (16:1/18:3) | 1.60 E-09 | 9.27 E-07 | 8.79 | 0.20 | Lipid | Phosphatidylcholine |
| 1-myristoyl-2-arachidonoyl-GPC (14:0/20:4) | 3.85 E-08 | 2.23 E-05 | 7.41 | 0.16 | Lipid | Phosphatidylcholine |
| 1-linoleoyl-2-linolenoyl-GPC (18:2/18:3) | 9.11 E-06 | 5.27 E-03 | 5.04 | 0.15 | Lipid | Phosphatidylcholine |
| Glycerophosphorylcholine | 5.89 E-06 | 3.40 E-03 | 5.23 | 0.12 | Lipid | Phospholipid Metabolism |
| 1-(1-enyl-stearoyl)-2-linoleoyl-GPE (P-18:0/18:2) | 1.98 E-07 | 1.14 E-04 | 6.70 | 0.13 | Lipid | Plasmalogen |
| 1-(1-enyl-palmitoyl)-2-linoleoyl-GPE (P-16:0/18:2) | 3.05 E-05 | 1.76 E-02 | 4.52 | 0.12 | Lipid | Plasmalogen |
| 1-(1-enyl-stearoyl)-2-oleoyl-GPE (P-18:0/18:1) | 6.92 E-06 | 4.00 E-03 | 5.16 | 0.11 | Lipid | Plasmalogen |
| Linolenate (18:3n3 or 3n6) | 1.02 E-06 | 5.91 E-04 | 5.99 | 0.16 | Lipid | Polyunsaturated Fatty Acid |
| Hexadecadienoate (16:2n6) | 2.18 E-05 | 1.26 E-02 | 4.66 | 0.14 | Lipid | Polyunsaturated Fatty Acid |
| Linoleate (18:2n6) | 4.41 E-05 | 2.55 E-02 | 4.36 | 0.12 | Lipid | Polyunsaturated Fatty Acid |
| 5alpha-pregnan-3beta,20alpha-diol monosulfate | 1.19 E-06 | 6.86 E-04 | 5.93 | 0.16 | Lipid | Progestin Steroids |
| Sphingomyelin (d18:2/14:0, d18:1/14:1) | 6.93 E-29 | 4.01 E-26 | 28.16 | 0.28 | Lipid | Sphingomyelin |
| Sphingomyelin (d17:2/16:0, d18:2/15:0) | 1.33 E-21 | 7.70 E-19 | 20.88 | 0.26 | Lipid | Sphingomyelin |
| Sphingomyelin (d17:1/14:0, d16:1/15:0) | 1.92 E-18 | 1.11 E-15 | 17.72 | 0.24 | Lipid | Sphingomyelin |
| Sphingomyelin (d18:2/21:0, d16:2/23:0) | 4.11 E-13 | 2.38 E-10 | 12.39 | 0.18 | Lipid | Sphingomyelin |
| Sphingomyelin (d18:1/21:0, d17:1/22:0, d16:1/23:0) | 3.76 E-10 | 2.17 E-07 | 9.43 | 0.17 | Lipid | Sphingomyelin |

**Supplementary Table S4. Metabolites significantly Increased in Women relative to Men over time**

**Supplementary Table S4. Metabolites significantly Increased in Women relative to Men over time (Continued)**

| **Metabolite** | **P-value** | **Bonferroni corrected P-value** | **-log10p** | **β Coefficient** | **Super Pathway** | **Sub Pathway** |
| --- | --- | --- | --- | --- | --- | --- |
| Sphingomyelin (d18:1/19:0, d19:1/18:0) | 1.28 E-09 | 7.38 E-07 | 8.89 | 0.15 | Lipid | Sphingomyelin |
| Sphingomyelin (d18:2/23:0, d18:1/23:1, d17:1/24:1) | 2.74 E-09 | 1.58 E-06 | 8.56 | 0.15 | Lipid | Sphingomyelin |
| Tricosanoyl sphingomyelin (d18:1/23:0) | 5.28 E-08 | 3.05 E-05 | 7.28 | 0.13 | Lipid | Sphingomyelin |
| Sphingomyelin (d18:1/25:0, d19:0/24:1, d20:1/23:0, d19:1/24:0) | 4.60 E-05 | 2.66 E-02 | 4.34 | 0.11 | Lipid | Sphingomyelin |
| Hexadecasphingosine (d16:1) | 3.29 E-05 | 1.90 E-02 | 4.48 | 0.13 | Lipid | Sphingosines |
| Gamma-glutamylglycine | 2.15 E-06 | 1.24 E-03 | 5.67 | 0.13 | Peptide | Gamma-glutamyl Amino Acid |

Note: Significant results presented following individual mixed effects modeling of each of the 578 individual metabolites measured at day 0, 3 and 7. All estimates adjusted for age, SAPS II, admission diagnosis, 25(OH)D at randomization, absolute change in 25(OH)D level at day 3 and plasma day (as the random-intercept). A multiple test-corrected threshold of P < 8.65 × 10^-5^ was used to identify all significant associations. GPC is Glycerophosphorylcholine; GPE is glycerophosphoethanolamine. Positive β coefficient values indicate higher metabolite abundance in females relative to males.

**Supplementary Table S5.** **Metabolites significantly decreased in Women relative to Men over time**

| **Metabolite** | **P-value** | **Bonferroni corrected P-value** | **-log10p** | **β Coefficient** | **Super Pathway** | **Sub Pathway** |
| --- | --- | --- | --- | --- | --- | --- |
| Alpha-ketoglutaramate | 2.67 E-06 | 1.54 E-03 | 5.57 | -0.13 | Amino Acid | Glutamate Metabolism |
| N-acetyl-aspartyl-glutamate | 4.83 E-09 | 2.79 E-06 | 8.32 | -0.20 | Amino Acid | Glutamate Metabolism |
| Pyroglutamine | 9.68 E-48 | 5.60 E-45 | 47.01 | -0.47 | Amino Acid | Glutamate Metabolism |
| N-acetylserine | 2.56 E-05 | 1.48 E-02 | 4.59 | -0.11 | Amino Acid | Glycine, Serine and Threonine Metabolism |
| N-acetylthreonine | 3.64 E-07 | 2.11 E-04 | 6.44 | -0.13 | Amino Acid | Glycine, Serine and Threonine Metabolism |
| Guanidinosuccinate | 3.62 E-06 | 2.09 E-03 | 5.44 | -0.20 | Amino Acid | Guanidino and Acetamido Metabolism |
| 1-methylguanidine | 2.17 E-07 | 1.25 E-04 | 6.66 | -0.24 | Amino Acid | Guanidino and Acetamido Metabolism |
| 4-guanidinobutanoate | 6.80 E-12 | 3.93 E-09 | 11.17 | -0.27 | Amino Acid | Guanidino and Acetamido Metabolism |
| 1-ribosyl-imidazoleacetate | 3.75 E-05 | 2.17 E-02 | 4.43 | -0.14 | Amino Acid | Histidine Metabolism |
| Imidazole lactate | 2.43 E-07 | 1.40 E-04 | 6.61 | -0.16 | Amino Acid | Histidine Metabolism |
| 1-methylhistidine | 4.93 E-07 | 2.85 E-04 | 6.31 | -0.16 | Amino Acid | Histidine Metabolism |
| 3-methylhistidine | 1.71 E-06 | 9.90 E-04 | 5.77 | -0.21 | Amino Acid | Histidine Metabolism |
| N-acetyl-1-methylhistidine | 3.44 E-07 | 1.99 E-04 | 6.46 | -0.22 | Amino Acid | Histidine Metabolism |
| Formiminoglutamate | 1.64 E-12 | 9.50 E-10 | 11.78 | -0.26 | Amino Acid | Histidine Metabolism |
| N-acetylcarnosine | 2.99 E-28 | 1.73 E-25 | 27.52 | -0.35 | Amino Acid | Histidine Metabolism |
| N-acetylvaline | 2.55 E-05 | 1.47 E-02 | 4.59 | -0.10 | Amino Acid | Leucine, Isoleucine and Valine Metabolism |
| N-acetylleucine | 7.65 E-06 | 4.42 E-03 | 5.12 | -0.12 | Amino Acid | Leucine, Isoleucine and Valine Metabolism |
| Alpha-hydroxyisocaproate | 9.27 E-07 | 5.36 E-04 | 6.03 | -0.13 | Amino Acid | Leucine, Isoleucine and Valine Metabolism |
| 3-hydroxyisobutyrate | 1.37 E-06 | 7.90 E-04 | 5.86 | -0.13 | Amino Acid | Leucine, Isoleucine and Valine Metabolism |
| 3-hydroxy-2-ethylpropionate | 2.06 E-09 | 1.19 E-06 | 8.69 | -0.15 | Amino Acid | Leucine, Isoleucine and Valine Metabolism |
| 2-hydroxy-3-methylvalerate | 5.00 E-08 | 2.89 E-05 | 7.30 | -0.16 | Amino Acid | Leucine, Isoleucine and Valine Metabolism |
| 2,3-dihydroxy-2-methylbutyrate | 4.77 E-07 | 2.76 E-04 | 6.32 | -0.17 | Amino Acid | Leucine, Isoleucine and Valine Metabolism |
| Beta-hydroxyisovalerate | 4.04 E-12 | 2.33 E-09 | 11.39 | -0.19 | Amino Acid | Leucine, Isoleucine and Valine Metabolism |
| N6,N6,N6-trimethyllysine | 3.62 E-13 | 2.09 E-10 | 12.44 | -0.20 | Amino Acid | Lysine Metabolism |
| Cysteine | 5.32 E-06 | 3.07 E-03 | 5.27 | -0.12 | Amino Acid | Methionine, Cysteine, SAM and Taurine Metabolism |
| 5-methylthioribose | 3.22 E-05 | 1.86 E-02 | 4.49 | -0.15 | Amino Acid | Methionine, Cysteine, SAM and Taurine Metabolism |
| S-adenosylhomocysteine | 2.23 E-07 | 1.29 E-04 | 6.65 | -0.16 | Amino Acid | Methionine, Cysteine, SAM and Taurine Metabolism |
| Lanthionine | 7.83 E-07 | 4.53 E-04 | 6.11 | -0.17 | Amino Acid | Methionine, Cysteine, SAM and Taurine Metabolism |
| Phenyllactate | 2.48 E-08 | 1.43 E-05 | 7.61 | -0.18 | Amino Acid | Phenylalanine Metabolism |
| N-acetylphenylalanine | 7.43 E-07 | 4.29 E-04 | 6.13 | -0.18 | Amino Acid | Phenylalanine Metabolism |
| Acisoga | 1.09 E-05 | 6.28 E-03 | 4.96 | -0.15 | Amino Acid | Polyamine Metabolism |
| (N(1) + N(8))-acetylspermidine | 6.12 E-07 | 3.53 E-04 | 6.21 | -0.16 | Amino Acid | Polyamine Metabolism |
| N-acetyl-isoputreanine | 3.87 E-06 | 2.24 E-03 | 5.41 | -0.16 | Amino Acid | Polyamine Metabolism |
| Indole-3-carboxylate | 4.60 E-06 | 2.66 E-03 | 5.34 | -0.13 | Amino Acid | Tryptophan Metabolism |
| Kynurenine | 5.46 E-08 | 3.16 E-05 | 7.26 | -0.14 | Amino Acid | Tryptophan Metabolism |
| Indoleacetate | 3.92 E-06 | 2.27 E-03 | 5.41 | -0.16 | Amino Acid | Tryptophan Metabolism |
| Picolinate | 2.51 E-07 | 1.45 E-04 | 6.60 | -0.20 | Amino Acid | Tryptophan Metabolism |
| N-acetylkynurenine | 3.00 E-06 | 1.74 E-03 | 5.52 | -0.22 | Amino Acid | Tryptophan Metabolism |
| 2-hydroxyphenylacetate | 6.82 E-06 | 3.94 E-03 | 5.17 | -0.16 | Amino Acid | Tyrosine Metabolism |
| 3-(4-hydroxyphenyl)lactate | 6.05 E-12 | 3.50 E-09 | 11.22 | -0.21 | Amino Acid | Tyrosine Metabolism |

**Supplementary Table S5.** **Metabolites significantly decreased in Women relative to Men over time (Continued)**

| **Metabolite** | **P-value** | **Bonferroni corrected P-value** | **-log10p** | **β Coefficient** | **Super Pathway** | **Sub Pathway** |
| --- | --- | --- | --- | --- | --- | --- |
| N-acetyltyrosine | 4.63 E-09 | 2.68 E-06 | 8.33 | -0.23 | Amino Acid | Tyrosine Metabolism |
| Argininate | 4.04 E-06 | 2.34 E-03 | 5.39 | -0.14 | Amino Acid | Urea cycle; Arginine and Proline Metabolism |
| Urea | 2.39 E-07 | 1.38 E-04 | 6.62 | -0.14 | Amino Acid | Urea cycle; Arginine and Proline Metabolism |
| Glucuronate | 2.27 E-06 | 1.31 E-03 | 5.64 | -0.18 | Carbohydrate | Aminosugar Metabolism |
| Sedoheptulose | 2.79 E-07 | 1.61 E-04 | 6.55 | -0.16 | Carbohydrate | Pentose Metabolism |
| Biliverdin | 1.64 E-05 | 9.48 E-03 | 4.79 | -0.14 | Cofactors and Vitamins | Hemoglobin and Porphyrin Metabolism |
| Bilirubin (E,Z or Z,E) | 2.07 E-08 | 1.20 E-05 | 7.68 | -0.18 | Cofactors and Vitamins | Hemoglobin and Porphyrin Metabolism |
| Bilirubin | 2.50 E-12 | 1.44 E-09 | 11.60 | -0.20 | Cofactors and Vitamins | Hemoglobin and Porphyrin Metabolism |
| D-urobilin | 2.26 E-06 | 1.31 E-03 | 5.65 | -0.20 | Cofactors and Vitamins | Hemoglobin and Porphyrin Metabolism |
| Nicotinamide riboside | 2.80 E-05 | 1.62 E-02 | 4.55 | -0.14 | Cofactors and Vitamins | Nicotinate and Nicotinamide Metabolism |
| 2-methylcitrate/homocitrate | 3.79 E-06 | 2.19 E-03 | 5.42 | -0.18 | Energy | TCA Cycle |
| Androstenediol (3beta,17beta) disulfate | 1.85 E-08 | 1.07 E-05 | 7.73 | -0.18 | Lipid | Androgenic Steroids |
| 5alpha-androstan-3beta,17alpha-diol disulfate | 1.88 E-11 | 1.09 E-08 | 10.73 | -0.27 | Lipid | Androgenic Steroids |
| 5alpha-androstan-3beta,17beta-diol disulfate | 3.92 E-27 | 2.27 E-24 | 26.41 | -0.36 | Lipid | Androgenic Steroids |
| 5alpha-androstan-3alpha,17beta-diol disulfate | 2.00 E-27 | 1.16 E-24 | 26.70 | -0.46 | Lipid | Androgenic Steroids |
| Deoxycarnitine | 8.43 E-14 | 4.87 E-11 | 13.07 | -0.19 | Lipid | Carnitine Metabolism |
| Propionylcarnitine (C3) | 6.88 E-05 | 3.98 E-02 | 4.16 | -0.12 | Lipid | Acylcarnitine |
| Malonylcarnitine (C3-DC) | 8.56 E-07 | 4.95 E-04 | 6.07 | -0.14 | Lipid | Acylcarnitine |
| Succinylcarnitine (C4) | 1.22 E-08 | 7.07 E-06 | 7.91 | -0.17 | Lipid | Acylcarnitine |
| 2-methylmalonylcarnitine (C4-DC) | 1.02 E-07 | 5.89 E-05 | 6.99 | -0.20 | Lipid | Acylcarnitine |
| Isovalerylcarnitine (C5) | 1.49 E-06 | 8.59 E-04 | 5.83 | -0.16 | Lipid | Acylcarnitine |
| 2-methylbutyroylcarnitine (C5) | 3.91 E-11 | 2.26 E-08 | 10.41 | -0.23 | Lipid | Acylcarnitine |
| Tiglyl carnitine (C5) | 7.31 E-14 | 4.23 E-11 | 13.14 | -0.24 | Lipid | Acylcarnitine |
| Glutaroylcarnitine (C5) | 7.32 E-15 | 4.23 E-12 | 14.14 | -0.24 | Lipid | Acylcarnitine |
| 3-Methyladipoylcarnitine (C7-DC) | 5.51 E-07 | 3.18 E-04 | 6.26 | -0.18 | Lipid | Acylcarnitine |
| Octanoylcarnitine (C8) | 1.55 E-06 | 8.96 E-04 | 5.81 | -0.16 | Lipid | Acylcarnitine |
| Cis-4-decenoylcarnitine (C10:1) | 1.29 E-06 | 7.48 E-04 | 5.89 | -0.15 | Lipid | Acylcarnitine |
| Decanoylcarnitine (C10) | 2.19 E-08 | 1.27 E-05 | 7.66 | -0.18 | Lipid | Acylcarnitine |
| Laurylcarnitine (C12) | 6.99 E-07 | 4.04 E-04 | 6.16 | -0.15 | Lipid | Acylcarnitine |
| Octadecanedioylcarnitine (C18-DC) | 3.26 E-10 | 1.88 E-07 | 9.49 | -0.22 | Lipid | Acylcarnitine |
| Octadecenedioylcarnitine (C18:1-DC) | 1.05 E-08 | 6.08 E-06 | 7.98 | -0.25 | Lipid | Acylcarnitine |
| N-acetyl-2-aminooctanoate | 3.61 E-05 | 2.08 E-02 | 4.44 | -0.15 | Lipid | Fatty Acid, Amino |
| Hydroxy-3-carboxy-4-methyl-5-propyl-2-furanpropanoate | 6.74 E-05 | 3.90 E-02 | 4.17 | -0.14 | Lipid | Fatty Acid, Dicarboxylate |
| 2-hydroxyadipate | 1.97 E-05 | 1.14 E-02 | 4.71 | -0.17 | Lipid | Fatty Acid, Dicarboxylate |
| Heptenedioate (C7:1-DC) | 1.55 E-06 | 8.95 E-04 | 5.81 | -0.18 | Lipid | Fatty Acid, Dicarboxylate |
| 3-methyladipate | 8.00 E-07 | 4.63 E-04 | 6.10 | -0.19 | Lipid | Fatty Acid, Dicarboxylate |
| 3-carboxy-4-methyl-5-propyl-2-furanpropanoate | 5.59 E-09 | 3.23 E-06 | 8.25 | -0.22 | Lipid | Fatty Acid, Dicarboxylate |
| Myo-inositol | 3.90 E-06 | 2.26 E-03 | 5.41 | -0.16 | Lipid | Inositol Metabolism |
| Pregnen-diol disulfate | 5.80 E-13 | 3.35 E-10 | 12.24 | -0.23 | Lipid | Pregnenolone Steroids |
| Glycochenodeoxycholate | 1.59 E-06 | 9.18 E-04 | 5.80 | -0.20 | Lipid | Primary Bile Acid Metabolism |
| Taurocholate | 4.19 E-05 | 2.42 E-02 | 4.38 | -0.21 | Lipid | Primary Bile Acid Metabolism |

**Supplementary Table S5.** **Metabolites significantly decreased in Women relative to Men over time (Continued)**

| **Metabolite** | **P-value** | **Bonferroni corrected P-value** | **-log10p** | **β Coefficient** | **Super Pathway** | **Sub Pathway** |
| --- | --- | --- | --- | --- | --- | --- |
| Glycochenodeoxycholate glucuronide | 2.94 E-12 | 1.70 E-09 | 11.53 | -0.28 | Lipid | Primary Bile Acid Metabolism |
| Taurochenodeoxycholate | 2.20 E-08 | 1.27 E-05 | 7.66 | -0.28 | Lipid | Primary Bile Acid Metabolism |
| Glycochenodeoxycholate sulfate | 4.26 E-11 | 2.46 E-08 | 10.37 | -0.32 | Lipid | Primary Bile Acid Metabolism |
| Glycodeoxycholate sulfate | 4.01 E-06 | 2.32 E-03 | 5.40 | -0.23 | Lipid | Secondary Bile Acid Metabolism |
| Glycolithocholate sulfate | 5.82 E-09 | 3.36 E-06 | 8.24 | -0.27 | Lipid | Secondary Bile Acid Metabolism |
| Glycocholenate sulfate | 6.22 E-14 | 3.59 E-11 | 13.21 | -0.28 | Lipid | Secondary Bile Acid Metabolism |
| Taurolithocholate 3-sulfate | 3.11 E-12 | 1.80 E-09 | 11.51 | -0.34 | Lipid | Secondary Bile Acid Metabolism |
| Taurocholenate sulfate | 1.03 E-16 | 5.94 E-14 | 15.99 | -0.38 | Lipid | Secondary Bile Acid Metabolism |
| 3beta,7alpha-dihydroxy-5-cholestenoate | 1.57 E-05 | 9.10 E-03 | 4.80 | -0.12 | Lipid | Sterol |
| 3beta-hydroxy-5-cholestenoate | 7.45 E-09 | 4.30 E-06 | 8.13 | -0.16 | Lipid | Sterol |
| Urate | 1.95 E-07 | 1.13 E-04 | 6.71 | -0.13 | Nucleotide | Purine Metabolism |
| N1-methylinosine | 1.89 E-07 | 1.09 E-04 | 6.72 | -0.18 | Nucleotide | Purine Metabolism |
| Xanthosine | 1.99 E-07 | 1.15 E-04 | 6.70 | -0.18 | Nucleotide | Purine Metabolism |
| Adenosine | 7.16 E-07 | 4.14 E-04 | 6.15 | -0.15 | Nucleotide | Purine Metabolism |
| N6-carbamoylthreonyladenosine | 1.34 E-06 | 7.72 E-04 | 5.87 | -0.15 | Nucleotide | Purine Metabolism |
| N2,N2-dimethylguanosine | 3.68 E-05 | 2.13 E-02 | 4.43 | -0.14 | Nucleotide | Purine Metabolism |
| 3-methylcytidine | 1.84 E-06 | 1.06 E-03 | 5.73 | -0.13 | Nucleotide | Pyrimidine Metabolism |
| Orotate | 2.34 E-05 | 1.35 E-02 | 4.63 | -0.15 | Nucleotide | Pyrimidine Metabolism |
| Dihydroorotate | 9.20 E-06 | 5.32 E-03 | 5.04 | -0.16 | Nucleotide | Pyrimidine Metabolism |
| Orotidine | 7.51 E-12 | 4.34 E-09 | 11.12 | -0.37 | Nucleotide | Pyrimidine Metabolism |
| 5,6-dihydrothymine | 4.93 E-05 | 2.85 E-02 | 4.31 | -0.11 | Nucleotide | Pyrimidine Metabolism |
| Pseudouridine | 4.45 E-05 | 2.57 E-02 | 4.35 | -0.1 | Nucleotide | Pyrimidine Metabolism |
| 3-ureidopropionate | 6.93 E-07 | 4.00 E-04 | 6.16 | -0.19 | Nucleotide | Pyrimidine Metabolism |

Note: Significant results presented following individual mixed effects modeling of each of the 578 individual metabolites measured at day 0, 3 and 7. All estimates adjusted for age, SAPS II, admission diagnosis, 25(OH)D at randomization, absolute change in 25(OH)D level at day 3 and plasma day (as the random-intercept). A multiple test-corrected threshold of P < 8.65 × 10^-5^ was used to identify all significant associations. For the Acylcarnitine sub pathway: a capital C is followed by the number of carbons within the fatty acyl group attached to the carnitine. A colon followed by a number is one or more unsaturated carbons in the acylcarnitine ester (i.e. C10:1 is a monounsaturated C10 acylcarnitine). DC following the carbon number is a dicarboxylic acylcarnitine. GPE is glycerophosphoethanolamine. Negative β coefficient values indicate lower metabolite abundance in females relative to males.

**Supplementary Table S6. Metabolites Differentially associated in women and men with 28-day mortality over time**

| **Metabolite** | **Bonferroni corrected P-value in women** | **Odds Ratio for 28-day mortality in women** | **Bonferroni corrected P-value in men** | **Odds Ratio for 28-day mortality in men** | **Super Pathway** | **Sub Pathway** |
| --- | --- | --- | --- | --- | --- | --- |
| Creatine | 1.00 | 2.48 | **3.67 E-06** | 2.99 | Amino Acid | Creatine Metabolism |
| N-acetylglutamine | 4.98 E-01 | 2.28 | **4.53 E-04** | 2.44 | Amino Acid | Glutamate Metabolism |
| cys-gly, oxidized | **1.59 E-04** | 4.02 | 1.00 | 1.45 | Amino Acid | Glutathione Metabolism |
| N-acetylhistidine | 1.00 | 1.93 | **2.65 E-07** | 3.60 | Amino Acid | Histidine Metabolism |
| 1-methylhistidine | **9.88 E-03** | 2.72 | 1.00 | 1.52 | Amino Acid | Histidine Metabolism |
| 1-ribosyl-imidazoleacetate | 1.00 | 1.43 | **4.72 E-03** | 2.13 | Amino Acid | Histidine Metabolism |
| Ethylmalonate | **3.36 E-05** | 4.03 | 6.54 E-01 | 1.85 | Amino Acid | Leucine, Isoleucine and Valine Metabolism |
| 2,3-dihydroxy-2-methylbutyrate | **2.78 E-03** | 3.53 | 1.00 | 1.63 | Amino Acid | Leucine, Isoleucine and Valine Metabolism |
| 3-hydroxyisobutyrate | **6.05 E-03** | 3.56 | 7.74 E-01 | 1.80 | Amino Acid | Leucine, Isoleucine and Valine Metabolism |
| Isovalerylglycine | 7.20 E-02 | 1.98 | **1.40 E-06** | 2.43 | Amino Acid | Leucine, Isoleucine and Valine Metabolism |
| N,N,N-trimethyl-5-aminovalerate | **9.67 E-04** | 3.45 | 1.00 | 1.72 | Amino Acid | Lysine Metabolism |
| Pipecolate | 1.00 | 1.99 | **2.30 E-03** | 2.33 | Amino Acid | Lysine Metabolism |
| Lanthionine | 5.26 E-01 | 1.94 | **5.72 E-04** | 2.22 | Amino Acid | Methionine, Cysteine, SAM and Taurine Metabolism |
| N-acetylputrescine | 4.38 E-01 | 2.34 | **9.93 E-08** | 3.32 | Amino Acid | Polyamine Metabolism |
| Prolylhydroxyproline | 1.00 | 1.05 | **1.17 E-02** | 2.61 | Amino Acid | Urea cycle; Arginine and Proline Metabolism |
| N-acetylcitrulline | 1.00 | 1.72 | **5.64 E-06** | 2.21 | Amino Acid | Urea cycle; Arginine and Proline Metabolism |
| N-acetylglucosaminylasparagine | **1.03 E-03** | 3.43 | 1.00 | 1.41 | Carbohydrate | Aminosugar Metabolism |
| Ribulonate | **3.67 E-03** | 2.78 | 1.00 | 1.54 | Carbohydrate | Pentose Metabolism |
| 1-methylnicotinamide | 1.00 | 1.53 | **1.40 E-09** | 3.17 | Cofactors and Vitamins | Nicotinate and Nicotinamide Metabolism |
| Nicotinamide riboside | 1.00 | 1.95 | **4.60 E-12** | 3.47 | Cofactors and Vitamins | Nicotinate and Nicotinamide Metabolism |
| N1-Methyl-2-pyridon E-5-carboxamide | 1.00 | 2.01 | **1.82 E-05** | 2.89 | Cofactors and Vitamins | Nicotinate and Nicotinamide Metabolism |
| Isobutyrylcarnitine (C4) | **3.22 E-06** | 3.77 | 1.25 E-01 | 1.66 | Lipid | Acylcarnitine |
| Glutaroylcarnitine (C5) | **1.34 E-03** | 3.26 | 1.89 E-01 | 1.89 | Lipid | Acylcarnitine |
| Adipoylcarnitine (C6-DC) | **2.13 E-05** | 3.31 | 1.00 | 1.32 | Lipid | Acylcarnitine |
| 3-methyladipoylcarnitine (C7-DC) | **1.56 E-04** | 3.26 | 1.00 | 1.33 | Lipid | Acylcarnitine |
| Suberoylcarnitine (C8-DC) | **1.73 E-06** | 3.23 | 1.00 | 1.44 | Lipid | Acylcarnitine |
| Decanoylcarnitine (C10) | **2.02 E-02** | 2.86 | 5.82 E-02 | 1.85 | Lipid | Acylcarnitine |
| N-palmitoyl-heptadecasphingosine (d17:1/16:0) | 1.00 | 1.27 | **2.53 E-02** | 2.34 | Lipid | Ceramide |
| Glycosyl-N-(2-hydroxynervonoyl)-sphingosine (d18:1/24:1(2OH)) | 1.00 | 0.73 | **9.41 E-03** | 2.48 | Lipid | Ceramide |
| Methylmalonate | **3.18 E-03** | 3.25 | 9.20 E-02 | 1.99 | Lipid | Fatty Acid Metabolism |
| 9,10-DiHOME | **3.57 E-02** | 2.49 | 5.92 E-02 | 1.84 | Lipid | Fatty Acid Metabolism |
| Pregnen-diol disulfate | **5.27 E-05** | 4.02 | 1.00 | 1.61 | Lipid | Pregnenolone Steroid |
| Androstenediol (3beta,17beta) disulfate | **4.33 E-03** | 3.16 | 1.00 | 1.46 | Lipid | Androgenic Steroids |
| 5alpha-pregnan-3beta,20alpha-diol disulfate | **3.85 E-03** | 2.49 | 7.45 E-02 | 1.82 | Lipid | Progestin Steroid |
| Glycochenodeoxycholate | 1.00 | 1.52 | **3.98 E-05** | 1.93 | Lipid | Primary Bile Acid Metabolism |
| Glycocholate | 8.18 E-01 | 1.65 | **6.96 E-05** | 1.79 | Lipid | Primary Bile Acid Metabolism |
| Tauroursodeoxycholate | 1.00 | 1.44 | **4.61 E-03** | 1.62 | Lipid | Secondary Bile Acid Metabolism |

**Supplementary Table S6. Metabolites Differentially associated in women and men with 28-day mortality over time (Continued)**

| **Metabolite** | **Bonferroni corrected P-value in women** | **Odds Ratio for 28-day mortality in women** | **Bonferroni corrected P-value in men** | **Odds Ratio for 28-day mortality in men** | **Super Pathway** | **Sub Pathway** |
| --- | --- | --- | --- | --- | --- | --- |
| Glycodeoxycholate sulfate | 5.36 E-02 | 1.91 | **3.56 E-05** | 1.71 | Lipid | Secondary Bile Acid Metabolism |
| Glycolithocholate sulfate | 1.00 | 1.64 | **8.35 E-05** | 1.77 | Lipid | Secondary Bile Acid Metabolism |
| Taurolithocholate 3-sulfate | 3.18 E-01 | 1.89 | **1.42 E-07** | 1.91 | Lipid | Secondary Bile Acid Metabolism |
| Campesterol | 1.00 | 1.13 | **2.64 E-05** | 3.40 | Lipid | Sterol |
| Beta-sitosterol | 9.46 E-02 | 2.20 | **1.66 E-06** | 2.73 | Lipid | Sterol |
| Adenosine | **2.03 E-02** | 2.97 | 1.00 | 1.68 | Nucleotide | Purine Metabolism |
| 3-methylcytidine | **1.43 E-02** | 3.67 | 1.00 | 1.09 | Nucleotide | Pyrimidine Metabolism |
| 3-ureidopropionate | 8.86 E-02 | 2.35 | **7.46 E-08** | 2.57 | Nucleotide | Pyrimidine Metabolism |

Note: Divergent results presented following individual mixed effects modeling of each of the 578 individual metabolites measured at day 0, 3 and 7 in women (N=151) and in men (N=277). All estimates adjusted for age, SAPS II, admission diagnosis, 25(OH)D at randomization, absolute change in 25(OH)D level at day 3 and plasma day (as the random-intercept). A multiple test-corrected threshold of P < 8.65 × 10^-5^ (Bonferroni corrected P-value < 0.05) was used to identify all significant associations shown in bold. For the Acylcarnitine sub pathway: a capital C is followed by the number of carbons within the fatty acyl group attached to the carnitine. DC following the carbon number is a dicarboxylic acylcarnitine. Odds Ratio = exp(β coefficient); β coefficient = ln(Odds Ratio).

**Supplementary Table S7. Day 3 Sex-specific Metabolic Networks with similar effects via Gaussian graphical models**

| **Module** | **Module P- value** | **Metabolite** | **Super Pathway** | **Sub-pathway** | **Component P-value** | **Component β Coefficient** |
| --- | --- | --- | --- | --- | --- | --- |
| **A** | **2.67 E-05** | 1-stearoyl-GPE (18:0) | Lipid | Lysophospholipid | **6.74 E-16** | 0.189 |
|  |  | 2-stearoyl-GPE (18:0) | Lipid | Lysophospholipid | **2.13 E-09** | 0.159 |
|  | | | | | | |
| **B** | **1.54 E-05** | 2-hydroxy-3-methylvalerate | Amino Acid | Leucine, Isoleucine and Valine Metabolism | **5.00 E-08** | -0.155 |
|  |  | Phenyllactate | Amino Acid | Phenylalanine Metabolism | **2.48 E-08** | -0.175 |
|  |  | 3-(4-hydroxyphenyl)lactate | Amino Acid | Tyrosine Metabolism | **6.05 E-12** | -0.213 |
|  |  | alpha-hydroxyisocaproate | Amino Acid | Leucine, Isoleucine and Valine Metabolism | **9.27 E-07** | -0.129 |
|  |  | 4-methyl-2-oxopentanoate | Amino Acid | Leucine, Isoleucine and Valine Metabolism | 6.33 E-03 | -0.066 |
|  | | | | | | |
| **C** | **1.15 E-05** | 3-hydroxy-2-ethylpropionate | Amino Acid | Leucine, Isoleucine and Valine Metabolism | **2.06 E-09** | -0.153 |
|  |  | beta-hydroxyisovalerate | Amino Acid | Leucine, Isoleucine and Valine Metabolism | **4.04 E-12** | -0.188 |
|  | | | | | | |
| **D** | **1.18 E-10** | 5alpha-androstan-3alpha,17beta-diol disulfate | Lipid | Androgenic Steroids | **2.00 E-27** | -0.464 |
|  |  | 5alpha-androstan-3beta,17beta-diol disulfate | Lipid | Androgenic Steroids | **3.92 E-27** | -0.364 |
|  | | | | | | |
| **E** | **8.86 E-06** | androstenediol (3alpha, 17alpha) monosulfate | Lipid | Androgenic Steroids | 1.49 E-04 | -0.143 |
|  |  | Pregnen-diol disulfate | Lipid | Pregnenolone Steroids | **5.80 E-13** | -0.228 |
|  |  | androstenediol (3beta,17beta) disulfate | Lipid | Androgenic Steroids | **1.85 E-08** | -0.181 |
|  | | | | | | |
| **F** | **1.20 E-08** | Glycochenodeoxycholate glucuronide | Lipid | Primary Bile Acid Metabolism | **2.94 E-12** | -0.277 |
|  |  | Glycochenodeoxycholate sulfate | Lipid | Primary Bile Acid Metabolism | **4.26 E-11** | -0.316 |
|  |  | Glycocholenate sulfate | Lipid | Secondary Bile Acid Metabolism | **6.22 E-14** | -0.277 |
|  |  | 3beta-hydroxy-5-cholestenoate | Lipid | Sterol | **7.45 E-09** | -0.163 |
|  |  | 3beta,7alpha-dihydroxy-5-cholestenoate | Lipid | Sterol | **1.57 E-05** | -0.123 |
|  |  | Taurocholenate sulfate | Lipid | Secondary Bile Acid Metabolism | **1.03 E-16** | -0.379 |
|  |  | Taurolithocholate 3-sulfate | Lipid | Secondary Bile Acid Metabolism | **3.11 E-12** | -0.337 |
|  | | | | | | |
| **G** | **1.63 E-05** | Palmitoleoyl-arachidonoyl-glycerol (16:1/20:4) | Lipid | Diacylglycerol | **3.80 E-03** | 0.102 |
|  |  | 1-palmitoleoyl-2-linolenoyl-GPC (16:1/18:3) | Lipid | Phosphatidylcholine | **1.60 E-09** | 0.197 |
|  |  | 1-palmitoleoyl-GPC (16:1) | Lipid | Lysophospholipid | **5.95 E-11** | 0.169 |
|  |  | 1-linolenoyl-GPC (18:3) | Lipid | Lysophospholipid | **1.57 E-11** | 0.195 |
|  |  | 1-arachidonoyl-GPC (20:4) | Lipid | Lysophospholipid | **8.26 E-07** | 0.126 |
|  |  | 1-arachidonoyl-GPE (20:4n6) | Lipid | Lysophospholipid | **2.74 E-10** | 0.162 |
|  |  | 1-linoleoyl-GPE (18:2) | Lipid | Lysophospholipid | **3.71 E-15** | 0.209 |

Note: Module P-value is the Bonferroni adjusted P-value of the GGM module; Metabolite is the Name of the metabolite in module; Super Pathway is the Name of the major biochemical pathway in the module; Sub-pathway is a subset of a the major biochemical pathway in the module; Component P-value and β coefficient results presented following individual mixed effects modeling of each of the 578 individual metabolites measured at day 0, 3 and 7. All estimates adjusted for age, SAPS II, admission diagnosis, 25(OH)D at randomization, absolute change in 25(OH)D level at day 3 and plasma day (as the random-intercept). A multiple test-corrected threshold of P < 8.65 × 10^-5^ was used to identify all significant associations shown in bold.

**Supplementary Table S8. Day 7 Sex-specific Metabolic Networks with similar effects via Gaussian graphical models**

| **Module** | **Module p value** | **Metabolite** | **Super Pathway** | **Sub-pathway** | **Component p value** | **Component β Coefficient** |
| --- | --- | --- | --- | --- | --- | --- |
| **H** | **1.34 E-05** | 2-hydroxy-3-methylvalerate | Amino Acid | Leucine, Isoleucine and Valine Metabolism | **5.00 E-08** | -0.155 |
|  |  | alpha-hydroxyisocaproate | Amino Acid | Leucine, Isoleucine and Valine Metabolism | **9.27 E-07** | -0.129 |
|  |  | Phenyllactate | Amino Acid | Phenylalanine Metabolism | **2.48 E-08** | -0.175 |
|  |  | 3-(4-hydroxyphenyl)lactate | Amino Acid | Tyrosine Metabolism | **6.05 E-12** | -0.213 |
|  |  | 4-methyl-2-oxopentanoate | Amino Acid | Leucine, Isoleucine and Valine Metabolism | 6.33 E-03 | -0.066 |
|  |  | alpha-hydroxyisovalerate | Amino Acid | Leucine, Isoleucine and Valine Metabolism | 4.62 E-04 | -0.098 |
|  |  | Phenylpyruvate | Amino Acid | Phenylalanine Metabolism | 2.03 E-02 | -0.066 |
|  | | | | | | |
| **I** | **2.66 E-07** | 3beta,7alpha-dihydroxy-5-cholestenoate | Lipid | Sterol | **1.57 E-05** | -0.123 |
|  |  | Glycolithocholate sulfate | Lipid | Secondary Bile Acid Metabolism | **5.82 E-09** | -0.275 |
|  |  | Taurolithocholate 3-sulfate | Lipid | Secondary Bile Acid Metabolism | **3.11 E-12** | -0.337 |
|  |  | Glycocholenate sulfate | Lipid | Secondary Bile Acid Metabolism | **6.22 E-14** | -0.277 |
|  |  | Taurocholenate sulfate | Lipid | Secondary Bile Acid Metabolism | **1.03 E-16** | -0.379 |
|  |  | Glycochenodeoxycholate glucuronide | Lipid | Primary Bile Acid Metabolism | **2.94 E-12** | -0.277 |
|  |  | Glycodeoxycholate sulfate | Lipid | Secondary Bile Acid Metabolism | **4.01 E-06** | -0.235 |
|  |  | 3beta-hydroxy-5-cholestenoate | Lipid | Sterol | **7.45 E-09** | -0.163 |
|  |  | Taurodeoxycholate | Lipid | Secondary Bile Acid Metabolism | 5.40 E-03 | -0.131 |
|  |  | Glycochenodeoxycholate sulfate | Lipid | Primary Bile Acid Metabolism | **4.26 E-11** | -0.316 |
|  | | | | | | |
| **J** | **4.93 E-05** | 3-hydroxy-2-ethylpropionate | Amino Acid | Leucine, Isoleucine and Valine Metabolism | **2.06 E-09** | -0.153 |
|  |  | 3-hydroxyisobutyrate | Amino Acid | Leucine, Isoleucine and Valine Metabolism | **1.37 E-06** | -0.134 |
|  |  | beta-hydroxyisovalerate | Amino Acid | Leucine, Isoleucine and Valine Metabolism | **4.04 E-12** | -0.188 |
|  | | | | | | |
| **K** | **2.27 E-05** | 3-methylcytidine | Nucleotide | Pyrimidine Metabolism | **1.84 E-06** | -0.133 |
|  |  | Phenylacetate | Amino Acid | Phenylalanine Metabolism | 3.43 E-02 | -0.077 |
|  |  | Phenylacetylglutamine | Peptide | Acetylated Peptides | 1.51 E-03 | -0.119 |
|  | | | | | | |
| **L** | **2.06 E-10** | 5alpha-androstan-3alpha,17beta-diol disulfate | Lipid | Androgenic Steroids | **2.00 E-27** | -0.464 |
|  |  | 5alpha-androstan-3beta,17beta-diol disulfate | Lipid | Androgenic Steroids | **3.92 E-27** | -0.364 |
|  | | | | | | |
| **M** | **2.82 E-05** | 1-linolenoyl-GPC (18:3) | Lipid | Lysophospholipid | **1.57 E-11** | 0.195 |
|  |  | 1-palmitoleoyl-GPC (16:1) | Lipid | Lysophospholipid | **5.95 E-11** | 0.169 |
|  |  | Palmitoleoyl-linoleoyl-glycerol (16:1/18:2) | Lipid | Diacylglycerol | 1.68 E-02 | 0.068 |
|  |  | Diacylglycerol (16:1/18:2 [2], 16:0/18:3 [1]) | Lipid | Diacylglycerol | 4.74 E-02 | 0.059 |
|  |  | Ceramide (d18:1/14:0, d16:1/16:0) | Lipid | Ceramides | 3.26 E-02 | 0.058 |
|  |  | N-palmitoyl-heptadecasphingosine (d17:1/16:0) | Lipid | Ceramides | 4.04 E-04 | 0.095 |
|  | | | | | | |
| **N** | **1.33 E-06** | 1-linoleoyl-GPA (18:2) | Lipid | Lysophospholipid | **5.69 E-10** | 0.198 |
|  |  | 1-palmitoyl-GPA (16:0) | Lipid | Lysophospholipid | **1.77 E-11** | 0.205 |

Note: Module P-value is the Bonferroni adjusted P-value of the GGM module; Metabolite is the Name of the metabolite in module; Super Pathway is the Name of the major biochemical pathway in the module; Sub-pathway is a subset of a the major biochemical pathway in the module; Component P-value and β coefficient results presented following individual mixed effects modeling of each of the 578 individual metabolites measured at day 0, 3 and 7. All estimates adjusted for age, SAPS II, admission diagnosis, 25(OH)D at randomization, absolute change in 25(OH)D level at day 3 and plasma day (as the random-intercept). A multiple test-corrected threshold of P < 8.65 × 10^-5^ was used to identify all significant associations shown in bold.
