# Supplementary material for: Metabolomic differences between critically Ill women and men
Source: Sci Rep. 2021 Feb 17;11:3951. doi: 10.1038/s41598-021-83602-5 (PMC7889607; doi:10.1038/s41598-021-83602-5)
Supplement: Supplementary file 6 — Supplementary Legend. [file 41598_2021_83602_MOESM6_ESM.docx]

Supplementary Figure 1. Rain Plot of repeated measures metabolomics data (day 0, 3 and 7) in Women or Men relative to 28-day mortality. Correlations between individual metabolites at day 0, 3 or 7 and 28-day mortality were determined separately for Women or Men utilizing logistic regression models correcting for age, SAPS II, admission diagnosis, 25(OH)D at day 0 and for absolute change in 25(OH)D level at day 3. The magnitude of beta coefficient estimates (effect size) is shown by a color fill scale and the corresponding significance level (-log_10_(P-value)) is represented by size of the circle. The intensity of the red fill color represents an increase in effect size for that metabolite relative to 28-day mortality. The intensity of the blue fill color represents a decrease in effect size for that metabolite relative to 28-day mortality. Statistical significance is the multiple test-corrected threshold of log_10_(P-value) > 4.06 which is equivalent to P-value < 8.65 × 10^-5^. All respective effect sizes ((beta coefficient =ln(Odds Ratio)) and P-values can be found in tabular form in Supplementary Table 6.
